# The Effects of Bipolar Disorder Risk on a Mobile Phone Keystroke Dynamics Based Biomarker of Brain Age

**DOI:** 10.3389/fpsyt.2021.739022

**Published:** 2021-12-22

**Authors:** John Zulueta, Alexander Pantelis Demos, Claudia Vesel, Mindy Ross, Andrea Piscitello, Faraz Hussain, Scott A. Langenecker, Melvin McInnis, Peter Nelson, Kelly Ryan, Alex Leow, Olusola Ajilore

**Affiliations:** ^1^Department of Psychiatry, University of Illinois at Chicago, Chicago, IL, United States; ^2^Department of Psychology, University of Illinois at Chicago, Chicago, IL, United States; ^3^Department of Bioengineering, University of Illinois at Chicago, Chicago, IL, United States; ^4^Graduate College, University of Illinois at Chicago, Chicago, IL, United States; ^5^Department of Psychiatry, The University of Utah, Salt Lake City, UT, United States; ^6^Department of Psychiatry, University of Michigan, Ann Arbor, MI, United States; ^7^College of Engineering, University of Illinois at Chicago, Chicago, IL, United States

**Keywords:** digital biomarkers, bipolar disorder, brain age estimation, smartphone, digital phenotyping

## Abstract

**Background:** Research by our group and others have demonstrated the feasibility of using mobile phone derived metadata to model mood and cognition. Given the effects of age and mood on cognitive performance, it was hypothesized that using such data a model could be built to predict chronological age and that differences between predicted age and actual age could be a marker of pathology.

**Methods:** These data were collected via the ongoing BiAffect study. Participants complete the Mood Disorders Questionnaire (MDQ), a screening questionnaire for bipolar disorder, and self-reported their birth year. Data were split into training and validation sets. Features derived from the smartphone kinematics were used to train random forest regression models to predict age. Prediction errors were compared between participants screening positive and negative on the MDQ.

**Results:** Three hundred forty-four participants had analyzable data of which 227 had positive screens for bipolar disorder and 117 had negative screens. The absolute prediction error tended to be lower for participants with positive screens (median 4.50 years) than those with negative screens (median 7.92 years) (*W* = 508, *p* = 0.0049). The raw prediction error tended to be lower for participants with negative screens (median = −5.95 years) than those with positive screens (median = 0.55 years) (*W* = 1,037, *p*= 0.037).

**Conclusions:** The tendency to underestimate the chronological age of participants screening negative for bipolar disorder compared to those screening positive is consistent with the finding that bipolar disorder may be associated with brain changes that could reflect pathological aging. This interesting result could also reflect that those who screen negative for bipolar disorder and who engaged in the study were more likely to have higher premorbid functioning. This work demonstrates that age-related changes may be detected *via* a passive smartphone kinematics based digital biomarker.

## Introduction

The development of biomarkers has long been a goal for psychiatry with the hope that these biomarkers may be able to facilitate early detection, diagnosis, and treatment selection—moving the field closer to a paradigm of precision medicine (1, 2). Aging is a heterogenous process associated with increased risk of morbidity and mortality. It has been proposed that differences between biological age and chronological age may be indicative of pathology, and various phenomena have been investigated as potential aging biomarkers including telomere length (3), DNA methylation (4), and features derived from neuroimaging (5, 6).

In previous work, our group identified age associated effects on smartphone typing kinematics—specifically enhancement of the difference between midday typing speed and typing speed at the beginning and end of the day (7). These kinematic data were collected *via* the BiAffect platform which collects such data passively as participants use their smartphones in their day-to-day routines thus enabling the creation of ecologically valid and temporally associated markers of cognitive performance (8).

Bipolar disorder is a psychiatric disorder characterized by recurrent episodes of mood disturbances. It is associated with cognitive deficits during mood episodes, some of which remain during euthymia (9, 10). It has also been proposed that bipolar disorder may exacerbate age associated neuropathologic processes in a phenomenon-termed neuroprogression (11, 12). In this study we investigated the hypothesis that cognitive changes associated with the disorder would be detectable *via* changes in typing kinematics. To do this, we leveraged the BiAffect platform taking advantage of the open enrollment of the project to obtain a large, heterogenous sample. Rather than utilizing binary self-report of diagnosis to distinguish between healthy controls and participants with a bipolar spectrum disorder, we categorized participants based on screening status on the Mood Disorders Questionnaire (MDQ), a screening instrument for bipolar disorder (13) using standard cut-off scores with sensitivity of 61% and specificity of 88% (14). We then examined differences in the performance characteristics of age prediction between the groups to investigate smartphone kinematic based age prediction's utility as a digital biomarker.

## Methods

Data for this study was collected as part of the open science BiAffect project. This study began in March 2018 with enrollment open to all adults in the United States with an iOS based smartphone that supports the BiAffect app. As of the time of the writing of this manuscript, the study is ongoing. Its protocol has been approved by the University of Illinois at Chicago Institutional Review Board.

The data for this study was collected from March 2018 to February 2021. Subject enrollment and data collection were all performed within the BiAffect app. The app includes modules for participants to complete questionnaires and perform tasks designed to measure aspects of cognitive performance such as response inhibition, set shifting, and reaction time. Its core technology is a custom built keyboard designed to replace the default keyboard. This keyboard collects typing related metadata including the type of keypress event (alphanumeric, backspace, autocorrection, etc.) and the associated timestamp. It does not collect the actual alphanumeric content. These data are then securely uploaded to the study server.

Participants completed the mood disorders questionnaire (MDQ) a screening instrument for bipolar disorder (13). The MDQs were typically completed at study entry. The performance characteristics of this instrument vary based on the setting but in general has been estimated to have relatively high specificity of 88% and adequate sensitivity of 61% (14) with a cut score of ≥7. Participants also provided self-reports of whether they have a diagnosis of a bipolar spectrum disorder, their birth year, and their gender. Given the fact that MDQ performance characteristics are better characterized than the reliability of self-reported bipolar disorder diagnosis and given the high rates of participants not disclosing their diagnosis status, it was decided to utilize MDQ status as a feature of interest in our analysis.

Analysis was restricted to participants who had provided at least 12 weeks worth of typing data. This was determined by calculating the median number of keystrokes per day across the entire sample and then filtering accordingly.

### Data Processing and Feature Engineering

Each subject's typing data was tokenized into sessions by grouping together consecutive keystroke events which have differences in timestamps of <5 seconds. Metrics were calculated for each session and then summarized for each subject. For sample entropy calculations the following parameters were used: *m* = 2, *r* = 0.2 ^*^ the standard deviation and tau = 1. Table 1 includes a description of these features. This data processing was performed via the pandas package, version 1.2.4 (15) for Python (Version 3.8.3).

**Table 1:** Model features.

**Feature**	**Description**
Mean_keypresses_per_session	Mean number of keypresses per session
Median_keypresses_per_session	Median number of keypresses per session
Standard_deviation_keypress_per_session	Standard deviation of keypresses per session
Median_absolute_deviation_keypress_per_session	Median absolute deviation of keypresses per session
Mean_interkey_time_mean	Mean of mean of interkey times per session
Median_interkey_time_mean	Median of mean interkey times per session
Standard_deviation_interkey_time_mean	Standard deviation of mean interkey times per session
Median_absolute_deviation_interkey_time_mean	Median absolute deviation of mean interkey times per session
Mean_interkey_time_median	Mean of median of interkey times per session
Median_interkey_time_median	Median of median interkey times per session
Standard_deviation_interkey_time_median	Standard deviation of median interkey times per session
Median_absolute_deviation_interkey_time_median	Median absolute deviation of median interkey times per session
Mean_autocorrect_rate	Mean autocorrect rate per session (# of autocorrect events / total # of keystrokes per session)
Median_autocorrect_rate	Median autocorrect rate per session (# of autocorrect events / total # of keystrokes per session)
Standard_deviation_autocorrect_rate	Standard deviation of autocorrect rate per session (# of autocorrect events / total # of keystrokes per session)
Median_absolute_deviation_autocorrect_rate	Median absolute deviation of autocorrect rate per session (# of autocorrect events / total # of keystrokes per session)
Mean_backspace_rate	Mean backspace rate per session (# of backspace events / total # of keystrokes per session)
Median_backspace_rate	Median backspace rate per session (# of backspace events / total # of keystrokes per session)
Standard_deviation_backspace_rate	Standard deviation of backspace rate per session (# of backspace events / total # of keystrokes per session)
Median_absolute_deviation_backspace_rate	Median absolute deviation of backspace rate per session (# of backspace events / total # of keystrokes per session)
Mean_session_length	Mean length of sessions in seconds
Median_session_length	Median length of sessions in seconds
Standard_deviation_session_length	Standard deviation of length of sessions in seconds
Median_absolute_deviation_session_length	Median absolute deviation of length of sessions in seconds
Sample_entropy_keypress	Sample entropy of # of keypresses per sessions
Sample_entropy_interkey_time_mean	Sample entropy of mean interkey times per session
Sample_entropy_interkey_time_median	Sample entropy of median interkey times per session
Sample_entropy_autocorrect_rate	Sample entropy of autocorrect rate per session
Sample_entropy_backspace_rate	Sample entropy of backspace rate per session
Sample_entropy_session_length	Sample entropy of session length in seconds

### Model Training and Assessment

Data were split into training and validation sets (75:25). Because of the collinearity among many of the features and the relative robustness of random forest models to collinearity (16), random forest models were used. Random forest models consist of a collection of decision trees whose individual predictions are aggregated to make a single prediction. They are a popular analytic tool in bioinformatics given their ability to model complex interactions (17). Two random forest regression models were trained using the caret and randomForest packages for R (18, 19). The mtry parameter determines the number of features that will be available for use when splitting nodes during the training of the model's decision trees. The mtry parameter was selected via a grid search of values ranging from 1 to 30 features using 10-fold cross-validation with 3 repeats. The mtry value which minimized the Root Mean Square Error (RMSE) was selected as the value used in the final models. The models were constructed in a stepwise fashion with the first model including only typing related features, and the second model included all features from the first with the addition of gender and MDQ screening status.

Each model's performance was assessed using the validation set to calculate RMSE, Breiman's pseudo R-squared, and median absolute error. Differences in model performance testing were assessed using paired Wilcoxon tests of their absolute errors. Feature importance was assessed using out-of-bag changes in Mean Square Error (MSE). Accumulated Local Effects plots (ALE Plots) (20) were constructed for features which appeared important or interesting. These plots allow the visualization of the effect of individual features and the interaction of two features on the model's prediction. They are especially useful when features may be correlated. Differences within model performance between participants based on MDQ screen status were assessed using Wilcoxon tests comparing raw prediction error scores and absolute prediction error scores. All tests were two sided with a significance level of 0.05. Family-wise error rates were controlled using the Holm-Bonferroni method. All statistical testing was performed in R (Version 4.0.0).

## Results

A total of 344 participants met criteria for inclusion in this analysis: 117 with negative MDQ screens and 227 with positive screens. As summarized in Table 2, the group with positive screens tended to have fewer males (Fisher's Exact *p* = 0.0042) and be younger than the positive screen group (*W* = 15,887, *p* = 0.0028. Compared to participants with positive MDQ screens, participants with negative screens had a lower rate of reporting a diagnosis of bipolar disorder, a higher rate of reporting no history of bipolar disorder, and also provided no diagnosis history at a lower rate (all comparisons Fisher's Exact *p* < 0.001). The participants with negative screens tended to have lower MDQ scores comparted to those with positive screens (*W* = 23,322, *p* < 0.001) and have a greater total number of keypresses (*W* = 15,098, *p* = 0.037).

**Table 2:** Subject characteristics.

	**MDQ negative**	**MDQ positive**	
# of participants	117	227	
% not male	60%	75%	*p* = 0.0042
Age in years, mean (sd)	41 (16)	35 (11)	
Age in years, median (mad)	39 (16)	33 (12)	W = 15,887, *p* = 0.0028
Age (min, max)	(20, 88)	(18, 70)	
Self-reports history of diagnosis with bipolar spectrum disorder	24 (21%)	115 (51%)	*p* < 0.001
Self-reports no history of diagnosis with bipolar spectrum disorder	66 (56%)	29 (13%)	*p* < 0.001
Does not provide any information regarding diagnosis of bipolar spectrum disorder	27 (23%)	83 (37%)	*p* < 0.001
MDQ score, mean (sd)	6 (4)	12 (1)	*W* = 23,322, *p < 0.001*
Total keypresses, mean (sd)	37,027 (87,464)	36,381 (71,262)	
Total keypresses, median (mad)	7,600 (7,465)	12,043 (12,682)	*W* = 15,098, *p = 0.037*

**Figure 1:**
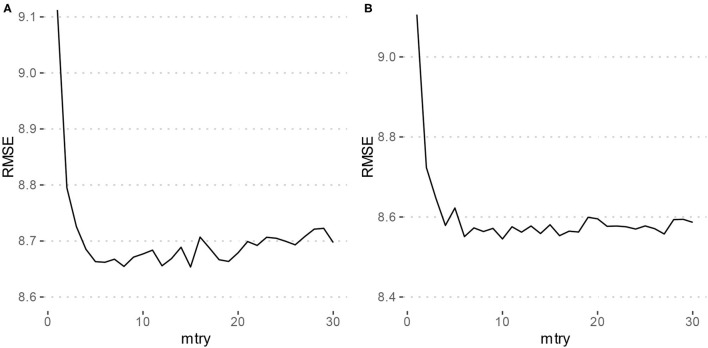
Parameter tuning results for random forest models. RMSE, Root mean squared error. **(A)** Depicts the grid search results for Model 1 (typing metrics only) which achieved a minimum RMSE at mtry = 15. **(B)** Depicts the grid search results for Model 2 (typing metrics with gender and MDQ status) which achieved a minimum RMSE at mtry = 10.

Using the criterion of minimizing RMSE for tuning the mtry parameter of the models, Model 1 which only included the typing metrics had an mtry = 15, and Model 2 which included the features of Model 1 as well as gender and MDQ status had an mtry = 10. Figure 1 depicts the tuning results.

Using the training set, both Model 1 and Model 2 had an RMSE of 8.7 years. The Breiman Pseudo R-squared values were 0.56 and 0.57 for Models 1 and 2, respectively. The performance of these models using the validation set are described in Table 3. Using the validation set, Model 1 had an RMSE of 9.7, and Model 2 had an RMSE of 9.5. Breiman's Pseudo R-squared was 0.42 and 0.44 for Models 1 and 2, respectively. Model 1 had a median absolute error of 5.9 and Model 2 had a median absolute error of 5.5. This difference was not statistically significant (*V* = 2,109, *p* = 0.21).

**Table 3:** Model performance comparison using the validation dataset.

	**Model 1 (only typing metrics)**	**Model 2 (typing metrics with MDQ status and gender)**	
RMSE	9.7	9.5	
Breiman's Pseudo R-Squared	0.42	0.44	
Median absolute error	5.9	5.5	*V* = 2,109, *p* = 0.21

Given the trend toward improved performance with the inclusion of gender and MDQ status as model features, analysis of feature importance and differences in prediction by MDQ status are presented only for Model 2.

**Figure 2:**
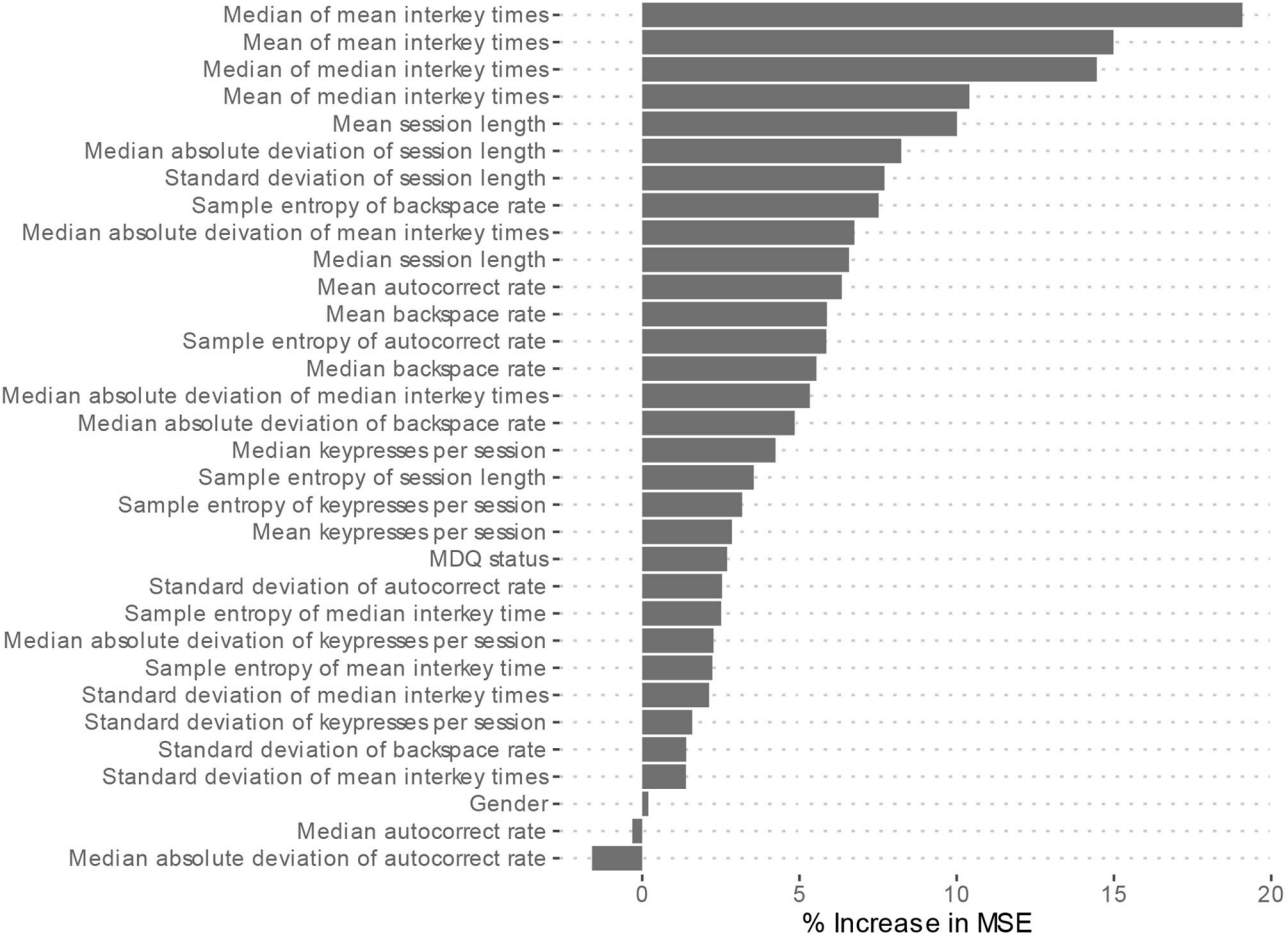
Model 2 Feature importance. MSE, Mean square error. Higher increases in MSE indicated increased importance of the feature in predicting age but do not indicate directionality of the relationship.

**Figure 3:**
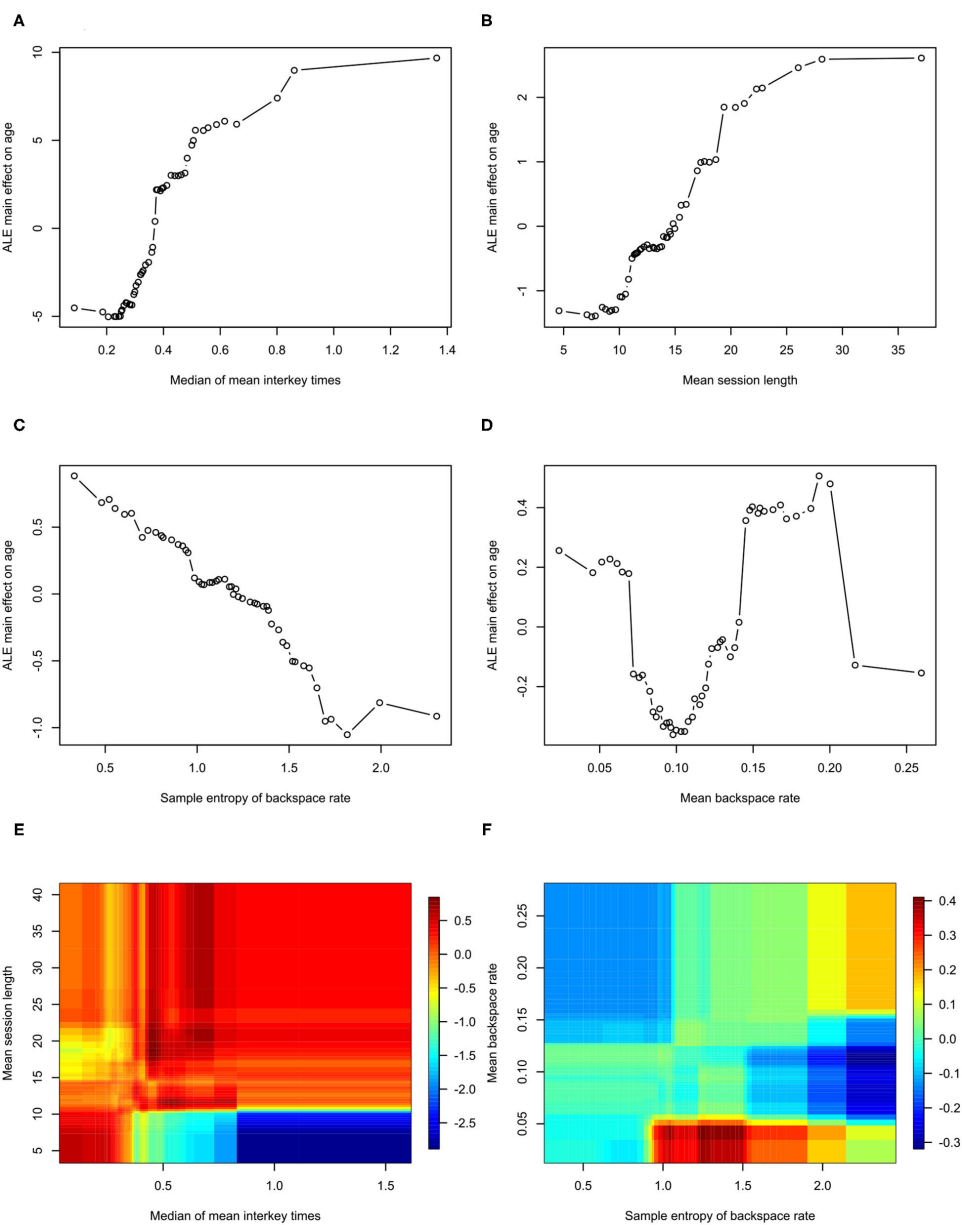
Accumulated Local Effects plots for Model 2. ALE, Accumulated Local Effects. **(A–D)** Depict the effects of individual features on age prediction. **(E,F)** Depict the interaction of the two indicated effects on age.

Feature importance is depicted in Figure 2. Features whose exclusion from the model results in a larger increase in Mean Squared Error are considered more important. While this allows us to understand the relative importance of the features, it does not provide information on how each feature's value is associated with age. One method that allows for the examination of these relationships in random forest models is an ALE plot. Given that many of the most important features are different summaries of the same essential feature (e.g., interkey time), in plots A–D of Figure 3 we present the ALE plots of four of the most important features: the median of mean interkey times, the mean session length, the sample entropy of the backspace rate, and the mean backspace rate. Based on these plots, increased interkey time and session length are both generally associated with increased age; whereas, increased sample entropy of the backspace rate is associated with younger age, and the association between age and the mean backspace rate is not monotonic. Plots E and F of Figure 3 depict the interaction between the median of mean interkey times and the mean session length and between the mean backspace rate and the sample entropy of the backspace rate, respectively. In these plots we see that the existence and directionality of linear trends between the predicted age and these features depend on the range of a second associated feature highlighting the complexity of the relationship between typing behaviors and predicted age.

**Figure 4:**
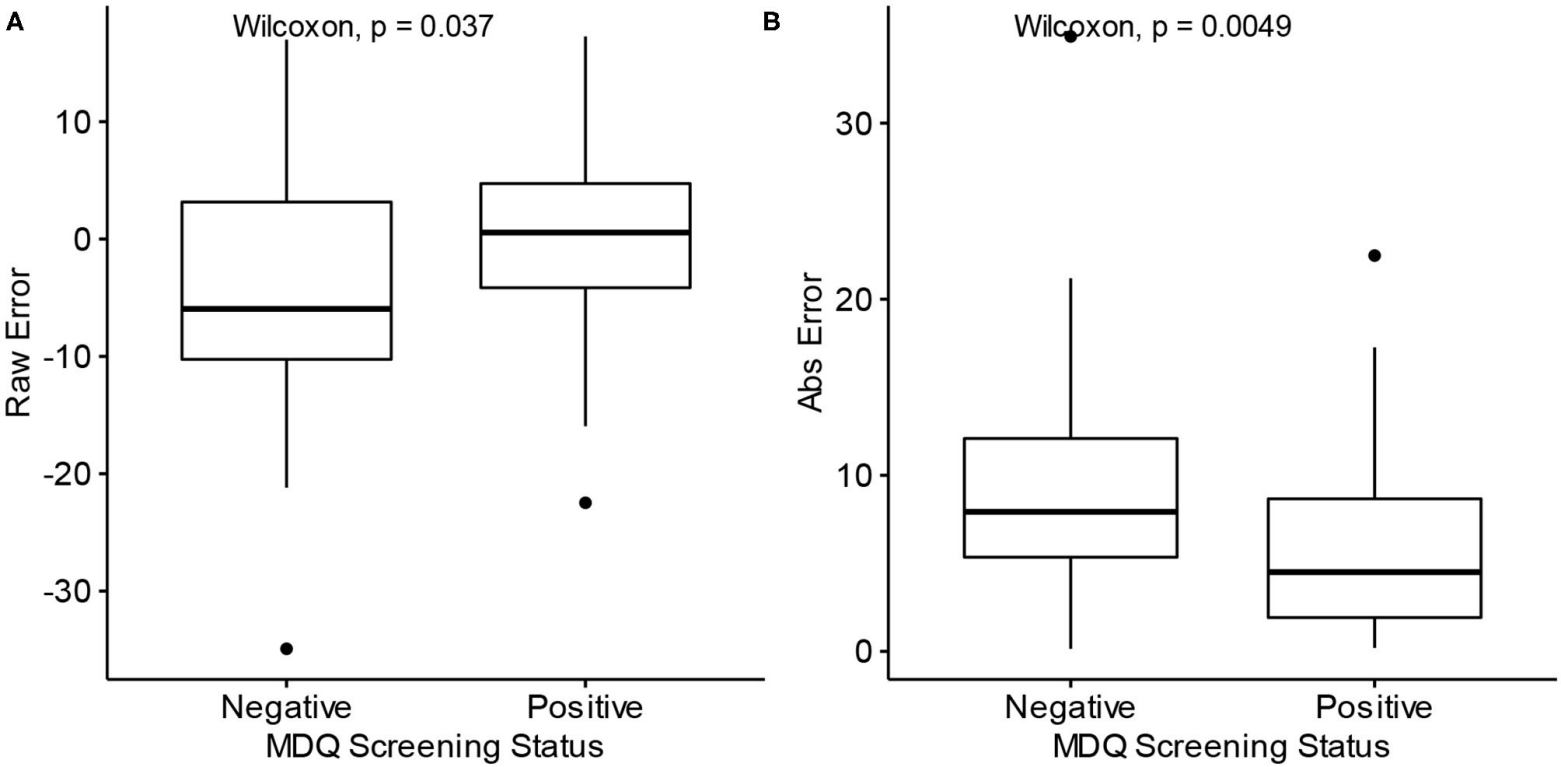
Differences in prediction error by MDQ status. MDQ, Mood disorder questionnaire. Abs Error, Absolute error. **(A)** Raw prediction errors of Model 2 by MDQ Screening status. **(B)** Absolute prediction errors of Model 2 by MDQ Screening status.

The raw prediction error for age, i.e., how many years over or under the model predicted from the correct age, tended to be lower for participants with a negative screen (median = −5.95) than those with positive screens (median = 0.55) (*W* = 1,037, *p* = 0.037). The absolute prediction error, which measures the absolute deviation from the correct age, tended to be lower for participants with a positive screen (median = 4.50) than those with negative screens (median = 7.92) (*W* = 508, *p* = 0.0049). These comparisons are depicted with boxplots in Figure 4. The significant difference in absolute prediction error between the groups suggests the existence of an intrinsic difference between the groups in terms of how each group's typing behaviors relate to age, and the significant difference in raw errors specifically demonstrates that participants with a negative MDQ screen tend to be predicted as younger may be consistent with the theory of bipolar disorder's association with neuroprogression.

## Discussion

Objective biomarkers of psychiatric pathology have the potential to transform the practice of psychiatry by providing clinicians more precise and reliable data to inform treatment decisions. In this study, we investigate the possibility of creating such a biomarker by using passively collected keyboard dynamics metadata derived from smartphone usage. This method of collection has the advantages of enabling high frequency sampling and perhaps even more importantly enabling such sampling to take place in people's normal day-to-day lives.

While there have been several studies which have used mobile phone derived metadata to predict demographic features such as age, these studies have tended to use features such as the number of calls and length of calls, the number of text messages, time of day of usage, and metrics derived from the networks of interactions (i.e., calls/text messages) between the users and other people (21–23). Further, these studies appear to have been focused on the utility of such methods for marketing applications, and in keeping with that aim, they used binned age groups and measured model performance based on correct classification of users to those groups. This makes comparing performance between these models and our regression oriented models difficult.

Although models of biological age are typically constructed by using a cohort of “healthy” participants to train a model which is then applied to participants with pathology, for this study we trained our model on both healthy and non-healthy participants in order to make maximum use of the available data. This is a limitation that we plan to address in future studies via larger, more well-characterized samples. The significant difference in prediction accuracy between participants with positive and negative MDQ screens suggests that there may be some intrinsic difference in the pattern of typing between participants who are likely at elevated risk of having bipolar disorder compared to those without such risk. That participants with positive screens have a lower absolute prediction error may be a consequence of the fact that the training sample consisted mostly of participants with positive screens; however, it may also be consistent with the emerging finding that psychiatric disorders may be characterized by a decrease in complexity and variability of behavior, which makes the brain less adaptable to a constantly changing environment (24). Even if the difference is primarily driven by the imbalance between participants with positive and negative MDQ screens, the very fact that such a difference exists is notable in that it suggests that the psychiatric pathology associated with a positive screen produces detectable changes in mobile phone typing kinematics. That there was also a tendency for the model to underpredict the age of participants with negative MDQ screens is consistent with the concept that pathology is associated with the phenomenon of biological age exceeding chronological age (5).

With this study design, we were not able to include in our models other potential covariates of cognitive and motor performance which could affect age prediction errors. Such factors could include co-morbidities, medication status, specific psychiatric diagnoses and severity, and general facility with phone usage. Given the tendency for bipolar disorder to be associated with a host of other co-morbidities (25), the between group differences found between participants with positive and negative MDQ screens could be due to bipolar disorder itself or some other combination of co-morbidities that is associated with people who are at increased risk of bipolar disorder that alters typing behaviors. Without such information, we cannot rule out the possibility that the differences in prediction accuracy between the participants with positive and negative MDQ screens are due to a disproportionate allocation of these characteristics which are not associated with the pathology associated with a positive MDQ screen. In future studies, we plan to collect data such as diagnoses, severity, and treatment status in order to create models which will properly attribute and quantify the effect of these variables on brain age biomarkers.

Examining which features are most important in predicting age, the most important features are measures of typing speed and the length of typing session. This is consistent with previous studies that have found correlations between age and typing performance (26–28). Plots A and B of Figure 3 demonstrate that both interkey time and session length tend to be positively correlated with age – older age is associated with slower speed and longer session; however, in examining the interaction of these two features depicted in plot D of Figure 3, we see that for sessions under 10 s in length, there is actually a negative correlation between interkey time and age. One possible explanation is that in these short sessions the interkey time represents the pauses that occur in a rapid exchange of text messages with another person, and that for longer sessions the interkey time represents the pauses that occur in the composition of a longer body of text.

The relatively high importance of the sample entropy of backspace rates across sessions is an intriguing finding. This feature theoretically measures the complexity of participant backspace use. Based on its ALE plot, plot E of Figure 3, it appears to generally negatively correlated with age; however, notably if we examine the interaction of the sample entropy of the rate with the overall mean rate depicted in plot D of Figure 3, we see that at relatively low overall usage of backspace increased entropy is associated with younger age, but that at relatively high usage increased entropy is associated with older age. Several studies have examined how measures of complexity like entropy can be applied to functional imaging derived brain networks and how complexity changes with aging (29–31). With data we are collecting in one of our current studies we will be able to examine how neuroimaging derived measures of brain complexity are associated with the complexity of signals derived from the kinematics of smartphone usage (32).

### Limitations

This study is limited by the fact that all subjective data were provided via self-report. A sample which consists of participants with clinically confirmed diagnoses would yield greater insight into the utility of digital biomarkers such as those described here in characterizing psychiatric disorders. Along those same lines, objective neuropsychological performance data would also have helped better contextualize our findings with existing literature in bipolar disorder. Although we included age and gender as features in our model, a sample in which age and gender distributions are equivalent across the case and control groups would likely yield more robust findings.

### Future Directions

Future directions of this research include attempting to replicate these findings in a more well-characterized sample and determining how this marker compares to other biomarkers such as neuroimaging based markers and other digital biomarkers. We also aim to investigate how differences in handedness (one-handed vs. two-handed typing) and distinguishing between different types of keystroke transitions (e.g., alphanumeric to alphanumeric vs. alphanumeric to backspace) might yield better performing models. Another potential line of investigation is determining whether differences between predicted age and chronological age by our model are associated with state level phenomena such as the severity of mood episodes.

## Conclusion

Passively collected typing kinematics can be used to estimate biomarkers of brain age. The differences we found in this study between the performance of such a biomarker in participants with and without a positive screen for bipolar disorder—i.e., the tendency to underestimate the age of healthy participants—suggest that this biomarker may also be a useful marker of pathology. Further investigation to refine the model and determine its relation to other markers of pathology such as neuroimaging and neuropsychological testing is warranted.

## Data Availability Statement

The original contributions presented in the study are included in the article/supplementary material, further inquiries can be directed to the corresponding author/s.

## Ethics Statement

The studies involving human participants were reviewed and approved by University of Illinois at Chicago Institutional Review Board. The patients/participants provided their written informed consent to participate in this study.

## Author Contributions

JZ did the analysis and wrote the manuscript. All other authors contributed data and edited the manuscript.

## Funding

This study was partially funded by Mood Challenge for Research kit (AL and PN) and 1R01MH120168 (OA and AL).

## Conflict of Interest

OA is a co-founder of KeyWIse AI. He also serves on the advisory boards of Embodied Labs and Blueprint Health. AL is an advisor for Buoy health and a consultant for Otsuka USA and ATAI Life Sciences, in addition to being a Co-founder of KeyWise AI. The remaining authors declare that the research was conducted in the absence of any commercial or financial relationships that could be construed as a potential conflict of interest.

## Publisher's Note

All claims expressed in this article are solely those of the authors and do not necessarily represent those of their affiliated organizations, or those of the publisher, the editors and the reviewers. Any product that may be evaluated in this article, or claim that may be made by its manufacturer, is not guaranteed or endorsed by the publisher.
